# Productivity and Time Use during Occupational Therapy and Nutrition/Dietetics Clinical Education: A Cohort Study

**DOI:** 10.1371/journal.pone.0044356

**Published:** 2012-08-31

**Authors:** Sylvia Rodger, Elizabeth Stephens, Michele Clark, Susan Ash, Cameron Hurst, Nicholas Graves

**Affiliations:** 1 Division of Occupational Therapy, School of Health and Rehabilitation Sciences, The University of Queensland, St. Lucia, Brisbane, Queensland, Australia; 2 School of Public Health, Queensland University of Technology, Kelvin Grove, Brisbane, Queensland, Australia; Penang Medical College, Malaysia

## Abstract

**Background:**

Currently in the Australian higher education sector higher productivity from allied health clinical education placements is a contested issue. This paper will report results of a study that investigated output changes associated with occupational therapy and nutrition/dietetics clinical education placements in Queensland, Australia. Supervisors’ and students’ time use during placements and how this changes for supervisors compared to when students are not present in the workplace is also presented.

**Methodology/Principal Findings:**

A cohort design was used with students from four Queensland universities, and their supervisors employed by Queensland Health. There was an increasing trend in the number of occasions of service delivered when the students were present, and a statistically significant increase in the daily mean length of occasions of service delivered during the placement compared to pre-placement levels.

**Conclusions/Significance:**

A novel method for estimating productivity and time use changes during clinical education programs for allied health disciplines has been applied. During clinical education placements there was a net increase in outputs, suggesting supervisors engage in longer consultations with patients for the purpose of training students, while maintaining patient numbers. Other activities were reduced. This paper is the first time these data have been shown in Australia and form a sound basis for future assessments of the economic impact of student placements for allied health disciplines.

## Introduction

Currently in the Australian higher education sector, a contested issue is whether the activity of supervising allied health students in clinical education placements is adequately compensated by the benefits. Global and contemporary evidence is sparse. Shortages of allied health clinical education placements have also resulted in a clinical education crisis [1], [2]. Changes in health/human services and higher education sectors that have limited allied health placement availability include reduced funding, shorter length of hospital stay, casualisation of the workforce and workforce shortages, lack of financial support to organisations and supervisors, and new models of care [2], [3]. Accentuating this problem is the proliferation of Australian allied health programs as well as increased quotas within existing programs. Given this challenging context, educators and practitioners alike are questioning the extent to which the costs of clinical education are adequately compensated by the benefits.

The aim of this paper was to describe productivity and time use changes from occupational therapy and nutrition/dietetics clinical education placements of students in Queensland, Australia as they are representative of the national variations seen across allied health practice. The information will inform economic arguments for allied health clinical education. This study did not attempt to estimate a cost benefit ratio for clinical education placements; that is a larger task. Quantifying changes to outputs and time use from clinical education based on sound research methods is an important step toward good policy making in health services and the tertiary sector.

Professional accrediting bodies for occupational therapy and nutrition/dietetics require different types of placement experiences. Dietitians undertake at least 10 weeks of individual case management placement as part of their accredited training [4]. Productivity and time use associated with the additional two types of nutrition/dietetic placements, food service and community public health nutrition, required in Australia are not reported in this paper as they are project-based. Project based dietetic placements, while impacting on community and population outcomes, do not readily translate into patient occasions of service. Within occupational therapy, students are required to undertake a variety of placements (with at least one of 8 weeks duration) that reflect the breadth of occupational therapy practice with people across the lifespan. Students work with people who have both recently acquired and long-standing health needs, with interventions that focus on the person, the occupation, and the environment [5]. This broad range of professional practice poses challenges in establishing a uniform research methodology to investigate productivity and time use.

Little is known about productivity changes during occupational therapy and nutrition/dietetics clinical education placements. Other health fields such as medicine have found it difficult to measure changes to service delivery outputs when students are present even though extensive tools for assessing a student’s clinical skills and knowledge have long been established [6]. The field of pharmacy has developed output measures tailored to their own discipline [7], [8], [9], [10]. A 2012 literature review investigated whether American students increased the number of pharmaceutical interventions administered to patients [7]. It concluded that it is cost saving to host a pharmacy clinical education placement and for the discipline of pharmacy, the productivity concept of ‘number of interventions’ is relevant and useful. However this service delivery output is too discipline-specific to be applied to productivity research in other allied health fields in Australia. A framework for the economic evaluation of clinical education placements has been proposed in Australia [11] and may be appropriate in a general context for assessing changes in costs and outputs for allied health by using Haines’ Quality Adjusted Passing Students Educated (QAPSE) outcome measure [11]. However, studies have still not identified an appropriate measure of outputs in allied health (that feed into an outcome measure), nor established reproducible methods to measure these output changes.

Some cost and benefit studies relevant to occupational therapy and nutrition/dietetics were conducted in the 1980 s and 1990 s in the United States and Canada and these studies appeared to stem from scrutiny by health care services regarding the costs to agencies of accommodating clinical education placements [12], [13], [14], [15], [16], [17], [18], [19], [20], [21], [22], [23]. These studies neglected the student perspective, failed to capture contemporary models of supervision or education practices beyond health care such as education, welfare, disability and the private sector, did not consider all costs and benefits, and were based on labour market values from over twenty years ago. The relevance of this information for current policy making is likely to be very limited.

In these previous studies, time use data were used as an indicator of costs and/or benefits but this was not often translated to service delivery outputs. One time use study reported a small net gain in workplace productivity when comparing full time equivalent additional staff time required during placements to students’ equivalent staff time in patient workload activities [12]. These results are only meaningful if it is assumed that students’ time on placement results in service delivery outputs.

Another difficulty with using time use data to measure allied health clinical education productivity is the risk of double-counting each team members’ contribution to an activity in a certain time period. To accurately measure students’ contribution to service delivery outputs without making assumptions about student competence, inclusion criteria need to be reported so that students’ passive, observational time is not counted towards overall productivity measures. Inclusion criteria were not reported in the Ladyshewsky studies [24], [25] but they weighted student productivity at both 100 and 60 per cent of supervisors’ productivity [25]. The 60 per cent weighted results assume that all students demonstrate a fraction of their supervisors’ competence, which may not always be true.

Measuring productivity and minimizing double-counting of team members’ time use has been employed by reporting the number of patients seen or treated [26], [27], [28]. Novel methods were used in one study that took a supervisor-student team perspective, and productivity changes were recorded with students present for four weeks and without students for four weeks [18]. The details of the clinical education sites were not provided and so any comparison with allied health practices in other jurisdictions was not possible without information about the settings studied. Measures of productivity beyond number of patients seen or treated also need to be established because of the importance of time spent in preparation for patients for example.

Much has changed in clinical education since the mid 1980 s and 1990 s, when most of the research on placements and productivity occurred [12], [13], [14], [15], [16], [17], [18], [19], [20], [21], [22], [23]. With these issues in mind, the research questions addressed in this study were: how do the number and the length of occasions of service delivered by the student-supervisor team change?; how do the patient care and non-patient care activities undertaken by the students and supervisors change during placements compared to before and after the placement when the students are not present in the organisation?; and how do students and supervisors use their time during clinical placements?

## Methods

### Ethics Statement

Ethics approval was granted by the Human Research Ethics Committees at Queensland Health, Mater Health Services, University of Queensland, Queensland University of Technology, Griffith University and James Cook University. Written informed consent was obtained from all participants involved in the study.

A cohort survey design with students from four Queensland universities, and their supervisors employed by Queensland Health between January and August 2010 was used.

Student participants were recruited from those allocated to final year clinical education placements in 2010. Supervisor participants were recruited from practicing nutritionist/dietitians and occupational therapists who had direct responsibility for student assessment in Queensland Health funded services. Placement models varied from one-to-one, to multiple students with multiple supervisors. Planned duration of student placements varied from 10 to 14 weeks for occupational therapy students and at least 10 weeks for nutrition/dietetics students at one or two different sites. Participants could join the study at any stage of the respective students’ placements, hence the number of participants for each week varied.

The survey was made available in electronic or paper form. For 30 minute blocks participants documented: how they spent their time according to particular time use categories (See Table 1); which patient they were managing, if relevant; and, whether they were working independently or with a supervisor or other student). Participants were allocated three random days out of a 5-day working week on which to complete the survey throughout the entire placement with students and supervisors allocated the same days. Supervisors were asked to complete the survey for the two weeks prior to placement commencement and for an additional two weeks post-placement. This provided data on pre- and post-placement time use. The dataset was organised for analysis using Microsoft Excel 2007 and statistical analysis undertaken with SPSS Version 18.

**Table 1:** Definitions of Time Use Categories for Students and Supervisors.

Patient Care Activities	
Direct patient care	Individual or group patient/client contact (member of the public); ward rounds; school visits; group-based therapy
Indirect patient care	Preparing for patient/client contact (member of the public); travel; documentation and discharge planning; managing patient issues; documentation and evaluation of patient/client contact; peer support; case conferences
**Placement Activities**	
Engaging in placement assessment	Placement reports; completing other assessment requirements
Managing the placement	Orientation; tuition; debriefs; feedback to student; communication with universities: not discussing specific patients/stakeholders
**Service Management**	Work unit meetings/communication eg. Emails; staff management/supervision; forms; human resource/payroll issues
**Other**	
Research (ethics approved)	Formal research project – leading or participating; completing this survey
Teaching and training – not related to the placement	Delivering in-service; guest lecture
Project interventions (no ethics approval required)	Primary prevention community interventions; community/stakeholder consultations; communication; peer support; partnership projects; consultancy work; reviewing workplace policies; undertaking quality improvement projects; audits; establishing evidence based practice
Project management processes	Reading literature; project preparation; report writing
Break	Paid or unpaid breaks eg. Morning tea
Undefined	Tasks not described above

Occasions of service were defined as the number of patients seen/managed in one day by the student-supervisor team and the length of an occasion of service was the number of minutes spent with/managing a patient by the student-supervisor team. The approach is unique to this study because time spent in indirect patient care activities such as maintaining patient records and travel for community visits is included in the definition of an occasion of service. Time use data for matched student-supervisor teams was translated to number and length of occasions of service to show joint team productivity. Inclusion criteria were established to make sure outputs could not exceed 100 per cent of service delivery capacity. This eliminated double counting of students’ and supervisors’ contribution and details are available from the authors.

Supervisors’ and students’ time use was calculated as independent daily means reported over the length of the placement. In the case where a student failed to report an activity but the supervisor did, then the supervisor response was used to augment the student dataset or vice versa. This only happened when at least 90 per cent of the working day could be inferred.

Mean daily number of occasions of service, length of occasions of service, and time use in minutes was reported as output measures. Outliers and low response data (less than 2 responses) from weeks 12 to 14 were removed to report number and length of occasions of service. The relationship between stage of placement (pre-, during and post-placement) and the various output indicators were modelled using a linear mixed modelling (LMM) approach [29]. This method was employed to capture the repeated measures structure of the observation and is more versatile than classical approaches of analysing repeated measures data. LMMs can deal with missing observations and are more versatile in implementing different and more appropriate residual covariance structures. A number of residual covariance structures were trialled in the LMM (CS:compound symmetry, AR(1) autoregressive order 1 US: Unstructured residual covariance structures) to obtain the most appropriate model [30].The CS residual covariance structure is that associated with the classical Univariate ANOVA approach to repeated measures analysis. CS assumes the between-subject variances are homogenous over time and that within-subject correlations do not vary regardless of the length of time intervening between measurements [29]. The AR(1) covariance structure also assumes between-subject variances are homogenous over time, but unlike the CS residual covariance structure, the degree of within subject covariance is allowed to decay as the intervening period between within-subject observations increases, albeit in a rather specific fashion [29]. Both the CS and AR(1) residual covariance structures only require the estimation of two parameters, regardless of the number of time points considered. For this reason CS and AR(1) are often preferable when sample sizes are small as they minimize the chance of over-fitting. However, the small number of parameters needed to estimate the CS and AR(1) covariance structures may not result in an error covariance structure that adequately fit the data. The US residual covariance matrix is the most flexible in that allows observations taken at different times to have heterogeneous variances, and within-subject correlations are allowed to differ (in any way) irrespective of the intervening time [29]. The disadvantage of the US residual covariance matrix is that it requires many parameters and models employing this covariance structure may be overfit, especially when sample sizes are small.

Assessing which covariance structure was most appropriate, and lead to the best model adequacy in general, was gauged using the standard approach for assessing Linear Mixed and Generalized Linear Models, namely deviance and Akaike’s Information Criteria (AIC) [31]. We tested if the overall effect of the stage of placement was significant, and conducted post-hoc t-tests for differences on the estimated marginal means resulting from the LMMs.

## Results

Of the potential cohort of students (N = 269) 34 students participated (12.6% response rate) and 47 of the potential cohort of 384 supervisors participated (12.2% response rate). The information in Table 2 shows the participants’ characteristics compared with estimated population data.

**Table 2:** Profile of Occupational Therapy and Nutrition/Dietetics Supervisors and Student respondent groups Compared to Queensland Workforce Population Data.

	Occupational Therapy andnutrition/dietetics participants	Occupational Therapy and nutrition/dietetics population estimates
**Supervisors’ age (n = 43)**		
≤34 years	72.1%	51.5%*
35≥54 years	18.6%	42.3%*
≥55 years	9.3%	6.3%*
**Supervisors’ gender (n = 43)**		
Female	95.4%	91.7%*
Male	4.7%	8.3%*
**Supervisors’ workplace location (n = 42)**		
Metropolitan	81.0%	62.7%?
Regional	16.7%	35.6%?
Remote	2.4%	2.2%?
**Supervisors who identify as CALD (n = 43)**	9.3%	NDA∼
**Number of students previously supervised (n = 43)**		
0–4	39.5%	NDA
5–10	14.0%	NDA
>10	46.5%	NDA
**Supervisors’ mean years full-time equivalent** **experience (n = 42)**	8.4 years (Range 1.5–26, SD 5.1)	NDA
**Students’ mean age (n = 27)**	21.8 years (Range 20–39, SD 5.1)	NDA
**Students’ gender (n = 27)**		
Female	81.5%	85.5%#
Male	18.5%	14.6%#

* Sourced from Brown, Capra, & Williams (2006) and Occupational Therapists Board of Queensland Annual Report 2008–09;

?Sourced from Brown, Capra, & Williams (2006) and university student placement databases;

# Sourced from university student placement databases;

∼ NDA = No data available.

**Figure 1:**
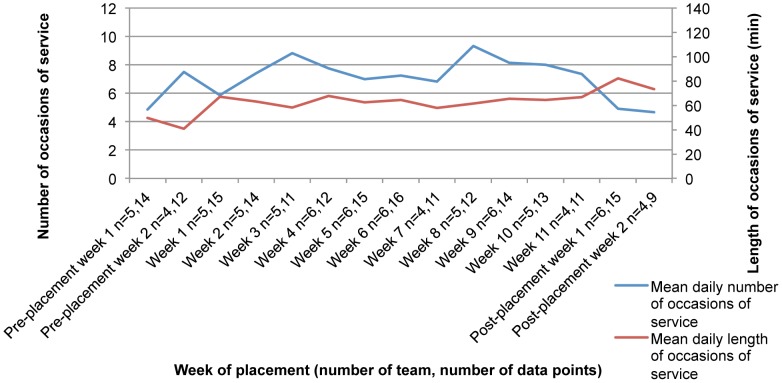
Occupational therapy and individual case management student-supervisor teams’ occasions of service, outliers removed. The blue line corresponds with the left axis showing how the number of occasions of service changes over the placement. The red line corresponds with the right axis showing how the length of occasions of services in minutes, changes. At each week of placement, we have provided the number of student-supervisor teams who responded to the survey, and the number of individual responses received from all teams.

**Figure 2:**
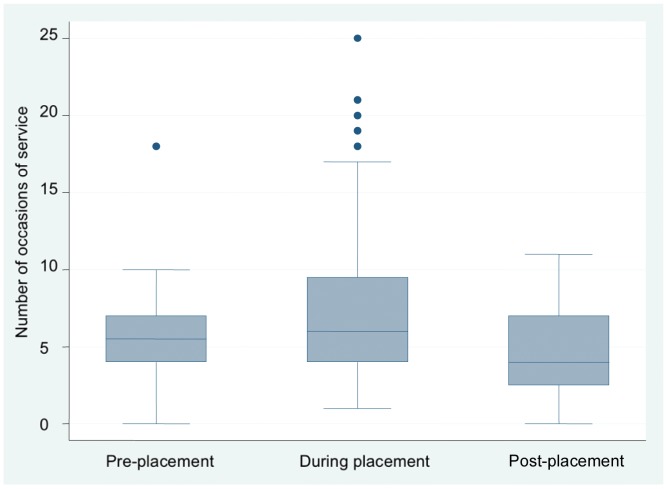
Occupational therapy and individual case management student- supervisor teams' daily number of occasions of service. This box and whisker plot shows the changing trend in student-supervisor teams’ number of occasions of service across the three time periods of interest.

**Figure 3:**
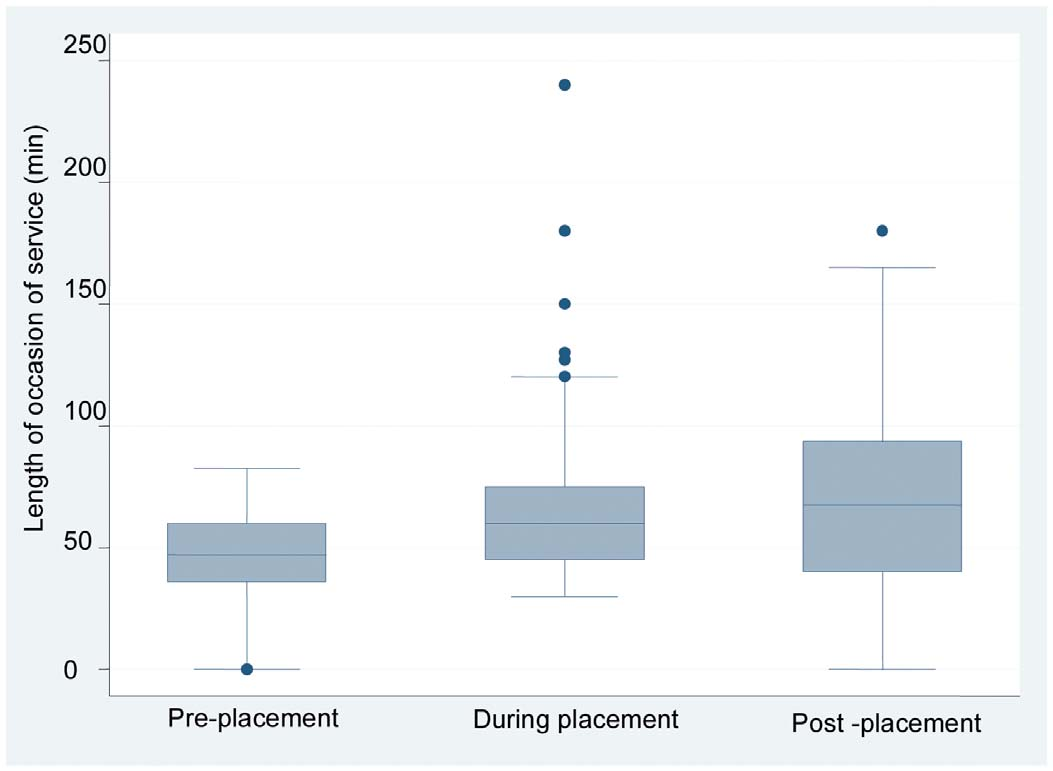
Occupational therapy and individual case management student- supervisor teams’ daily length of occasions of service. This box and whisker plot shows the changing trend in student-supervisor teams’ length of occasions of service across the three time periods of interest.

The mean daily number and length of occasions of service for occupational therapy and individual case management nutrition/dietetics student-supervisor teams during each week of placement are shown in Figure 1. In weeks 1 to 3, there was an increase in the number of occasions of service and a decrease in length of occasions of service compared to pre-placement. Minimal changes occurred in weeks 4 to 7 but number of occasions of service peaked in week 8. Number and length of occasions of service trended towards pre-placement levels after the students left the workplace. The information in Figure 2 shows the mean daily number of occasions of service increasing for the placement phase as compared to before and after. This result was not statistically significant (F = 0.202 _(2,19.281 df)_, p = 0.819). Figure 3 shows the length of occasions of service increasing over the three stages of placement. There was a significant increase in the mean daily length of occasions of service between pre- and during placement. Table 3 shows how mean daily number and length of occasion of service change over the three stages of placement. In the two models, we used the error covariance structure best fit (deviance and Akaike’s Information Criteria). For both number and length of occasions of service, the compound symmetry error covariance structure provided the most adequate model.

**Table 3:** Linear Mixed Model Results for Number and Length of Teams’ Occasions of Service.

Variable	Pre-placement Estimated Marginal Mean (95%Confidence Interval)	During placement Estimated Marginal Mean (95%Confidence Interval)	Post-placement EstimatedMarginal Mean(95% Confidence Interval)	Differing stages of placement
Number of occasions of service	5.8 (1.8∶9.7)	5.9 (3.5∶8.3)	7.1 (3.5∶10.7)	Model not significant
Length of occasions	56.1 (40.1∶72.1)	80.5 (69.6∶91.3)	72.5 (57.4∶87.5)	Pre < During p = 0.011*
of service (min)				Pre = Post p = 0.077
				During = Post p = 0.306

**Figure 4:**
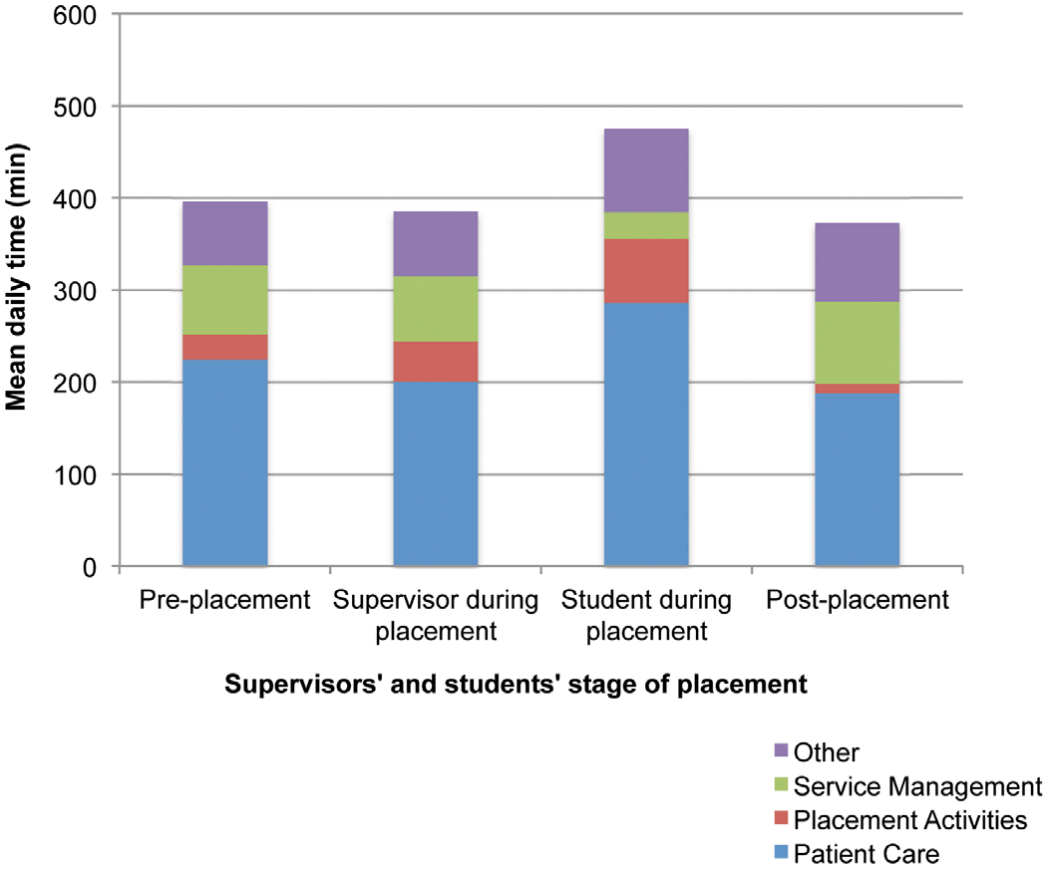
Occupational therapists, individual case management nutritionist/dietitians' and students' mean daily time spent in various activities. The proportion of time (minutes) spent in each of the four key time use categories is shown for occupational therapy and individual case management nutrition/dietetic supervisors pre-, during and post-placement, and for students during placement.

To investigate supervisor and student time use, data from the detailed time use categories in Table 1 were collapsed into the following major headings: patient care; placement activities; service management; and other. The information in Figure 4 shows supervisors’ and students' mean daily time spent in various activities across the three stages of placement (pre, during and post). Occupational therapy and dietetic supervisors’ time in patient activities decreased during placement from pre-placement levels, with presumably the students taking on more of this activity (See Figure 4). This is illustrated by students’ patient care time during placement being higher than that of their supervisors. Post-placement supervisors’ time spent in patient activities remained consistent and did not return to the pre-placement state during these two weeks. Post-placement, supervisors engaged in more service management activities.

In the various models used to examine differences in time use across the stages of placement, we again used the error covariance structure best fit (deviance and Akaike’s Information Criteria). For patient care, non-patient care and service management activities, the unstructured error covariance structure provided the most adequate model, whereas for placement activities the compound symmetry model was the most adequate.

The results of the linear mixed models showed a statistically significant difference between the daily mean supervisor time spent in patient care activities pre-placement and during placement (p = <0.001) (Table 4). For supervisor time spent in all non-patient care activities, post hoc analysis showed a significant difference between the daily mean supervisor time spent in non-patient care activities pre- and during placement (p = 0.002), and between during and post-placement (p = <0.001). For placements with a patient care focus, post hoc analysis of supervisor time spent in placement activities showed a significant difference between the daily mean supervisor time spent in placement activities during and post-placement (p = <0.001) (Table 4). The differences in mean daily time in service management activities were not significant for all types of placements.

**Table 4:** Linear Mixed Model Results for Selected Occupational Therapy and Nutrition/Dietetics Time use Variables.

Variable	Pre-placement Estimated Marginal Mean (95%Confidence Interval)	During placement Estimated Marginal Mean (95%Confidence Interval)	Post-placement Estimated Marginal Mean (95%Confidence Interval)	Differing stages of placement
Patient care activities	285.6	149.5	203.2	Pre > During p<0.001*
(min)	(232.9∶338.2)	(109.5∶189.5)	(144.0∶262.4)	Pre = Post p = 0.073
				During = Post p = 0.098
Non-patient care	84.7 (50.7∶118.6)	167.8	70.9 (35.7∶106.1)	Pre < During p = 0.002*
activities (min)		(135.2∶200.5)		Pre = Post p = 0.503
				During >Post p<0.001*
Placement activities	29.1 (15.0∶43.2)	43.0 (32.0∶54.1)	12.1	Pre = During p = 0.071
(min)			(-2.8∶27.0)	Pre = Post p = 0.065
				During <Post p<0.001*
Service management activities (min)	82.6 (66.2∶99.0)	73.4 (56.3∶90.5)	92.4 (63.1∶121.6)	Model not significant

* statistically significant at the 5% level.

Our data further describes time use week by week across the placement. Supervisors’ time was spent mostly in patient care activities, followed by service management. This was consistent across all the weeks surveyed including the two weeks pre- and post-placement. Their time spent in placement activities increased in the first few weeks of placements and again towards the end of placement during weeks 11 and 14. The majority of occupational therapy and dietetic students’ time was spent in patient care activities, with this increasing over the first few weeks of placement and peaking at weeks 5 to 6, and again at week 12 for those on longer placements. The second most common time use category for students was placement activities.

## Discussion

We investigated time use and productivity changes during occupational therapy and dietetic placements. The response rate was poor suggesting cautious interpretation of the findings. Outputs measured were number of occasions of service, length of occasions of service and minutes spent in various non-patient care related time use categories. Unpublished Australian reports have recommended that measures of productivity outputs other than number of patients seen or number of billable activities be used in studies such as these. In response, we collected supervisors’ and students’ independent time spent in non-patient care related activities. For these types of activities, allied health professionals have wide-ranging approaches to measuring outputs making it difficult to assess productivity beyond the patient care context. Beyond this, productivity outcomes directly associated with clinical education such as improved performance/functioning and independence or reducing nutrition-related chronic disease risk are difficult to measure.

There was a net increase in productivity outputs measured by daily mean number of occasions of service when the student was present in the workplace compared to pre- and post-placement indications of normal service delivery. These are similar results to Leiken et al. [27] who concluded that students had a positive impact on the productivity of hospital services defined by number of patient treatments per day. Dillon et al. [28] also found that student-supervisor teams saw 15 per cent more patients per day than supervisors alone. As expected, mean daily length of occasions of service significantly increased when students were present due to the patient related teaching undertaken. However, increased length of occasions of service continued after the student placement had ceased. We did not adjust for possible confounders beyond the clinical education program that may have affected these results. Nor did we identify the case-mix of the student-supervisor team and distinguish between new and continuing patients, which would be interesting to investigate in future studies. Supervisors worked with great diligence while hosting students on placement as suggested by the trend for both number and length of occasions of service to increase from pre- to during placement.

We found a fairly consistent 40 to 60 minutes per day of supervisor time spent in placement activities across the entire duration of the placement. We also saw a significant drop in the mean daily time spent in placement activities when the students left the placement. In contrast to our study, Chung and Spelbring [13] reported that a high number of staff instructional hours were needed in week one but over the course of the placement they dropped to four hours per week.

A major limitation of this study was that two weeks of data collection pre- and post-placement may not provide valid indications of ‘normal’ productivity for all supervisors. In particular, there may be a workload flow-on effect from the students’ presence post-placement. In terms of representativeness, the low response rate is also a major limitation to the study. However, this is one of the largest known studies of its kind and provides useful preliminary data for allied health professions. In any one week of the study, a maximum of two complete student-supervisor teams from the nutrition/dietetics domain provided useable data to measure number and length of occasions of service. As such, the productivity results presented in this paper are predominantly occupational therapy data. Although the study found similar patterns in productivity between occupational therapy and nutrition/dietetics, the nutrition/dietetics discipline should be aware of this limitation when interpreting the results.

We recommend this study be repeated with a larger sample of allied health students and supervisors. It is also recommended that for Australian studies, the Australian Health Classification System [32] time use categories be used in the future so that a consistent approach is applied nationally. Future research questions worthy of consideration include:

- How could other measures such as patient satisfaction or quality of student work/competence be used to evaluate productivity impacts of clinical education?
- Do students become more independent in their work over time on placement and what impact does this have on supervisor time use and productivity?
- Does the case-mix of new versus continuing patients and complexity of diagnostic related groups being serviced change during student placements and how does this affect productivity?
- What is an appropriate measure of productivity for allied health disciplines that do not work directly with patients?

In this study, we established a method for reporting productivity and time use changes during clinical education placements. Detailed time use data based on 30 minute intervals was collected for students and supervisors on three randomly-allocated working days throughout the entire placement. We developed two survey instruments one for students for completion during placements and one for supervisors for completion two weeks pre-, during placement, and two weeks post-placement.

Student-supervisor teams undertook more occasions of service when students are on placement, although this conclusion largely reflects occupational therapy data. Mean daily length of occasions of service increased significantly from pre- to during and continued increasing to post-placement. More students’ time was spent in patient care activities than any other category of time use followed by placement activities. This research will contribute to future assessments of the economic impact of student placements.
